# A Finite Element Analysis of Mandibular Anterior Intrusion Using Mini-Screws With Three Different Implant Insertion Angles

**DOI:** 10.7759/cureus.99879

**Published:** 2025-12-22

**Authors:** Abirami Vetriselvan, Saravanan Pichai, Revathi Peddu, Ashok Kumar Talapaneni, Devikanth Lanka, Aruna Dokku

**Affiliations:** 1 Department of Orthodontics and Dentofacial Orthopedics, Sibar Institute of Dental Sciences, Guntur, IND

**Keywords:** deep bite, finite element method, insertion angle, intrusion, mini-screw, primary implant stability, temporary anchorage devices, von mises stress

## Abstract

Background

Facial aesthetics is a growing concern among young individuals, which greatly depends on the underlying dentofacial harmony. Among the anomalies in the three-dimensional plane of space, the problems related to the vertical plane of dentition are the most arduous to treat. It is commonly associated with supra-eruption of anterior teeth that are frequently treated using mini-screws as a skeletal anchorage device. The various factors associated with the stability of mini-screws include the insertion angle of the mini-screw in effecting a true intrusion with uniform distribution of stress. This finite element study evaluated the von Mises stress generated on the periodontal ligament and displacement in labio-lingual and apical directions during the intrusion of four- and six- mandibular anterior teeth using bilateral mini-screws inserted at 90°, 60°, and 30° in relation to the bone surface directed apically.

Methodology

Six finite element models of mandibular four- and six- anterior teeth with bilateral mini-screws were simulated with insertion angles of 30°, 60°, and 90° with disto-intrusive force vectors. The von Mises stress and displacement along apical and labiolingual axes were assessed when the force levels were applied, and a non-linear static analysis was performed using ANSYS version 18.1 software.

Results

The highest von Mises stress was elicited at 30° insertion in both four-teeth (0.002028 MPa) and six-teeth (0.002856 MPa) intrusion scenarios, which decreased with 60° (0.001948 MPa and 0.002768 MPa, respectively) and the least at 90° (0.001885 MPa and 0.002675 MPa, respectively). The teeth showed maximum labial tipping at 90° (0.000353 mm in four-teeth and 0.000507 mm in six-teeth) and least at 30°. Maximum intrusion was elicited at 90°, followed by 60°, and the least at 30°. Central incisor underwent maximum intrusion in both four- and six-teeth scenarios.

Conclusions

The study findings suggest the oblique placement of bilateral mini-screws at around 60° with a disto-intrusive force vector to achieve a relatively true intrusion with optimal stress generation and minimal labial tip in four- and six- mandibular anterior teeth.

## Introduction

Over the past few decades, there has been a growing concern for facial esthetics, which has led to a paradigm shift in seeking orthodontic treatment for enhancing smile and profile esthetics rather than merely achieving functional efficiency of the dentofacial structures. Among the numerous dentoskeletal aberrations occurring in various planes, correction of vertical discrepancies is the most arduous task. The global burden of vertical disharmonies revealed an overwhelming prevalence of deep overbites, accounting for over 21.98 ± 14.13% in permanent dentition and 24.34 ± 14.54% in mixed dentition [1]. A vast number of these patients with deep overbite often benefit from lower incisor intrusion, as its display increases with age. The segmental arch technique by Burstone [2] and the bio-progressive therapy by Ricketts et al. [3] have been popularly used for anterior intrusion, obtaining the anchorage from posterior teeth. Nevertheless, this resulted in reactive extrusion of the posterior dentition. This necessitated the conception of an absolute anchorage system that offered complete resistance to movement, paving the way to the introduction of temporary anchorage devices for the correction of most malocclusions [4,5]. The use of mini-screws or mini-implants has been widely exploited as a stable anchorage unit in bringing about various tooth movements due to their small size, favoring their use in various anatomic sites, ease of placement, and cost efficiency [5].

The key factors influencing mini-implant stability include the implantation angle, which holds a prime biomechanical role due to its correlation with cortical bone contact. The implant’s angulation and resulting force vector significantly influence the periodontal ligament (PDL) stress and control the extent of three-dimensional (3D) displacement during lower anterior teeth intrusion by modulating the direction of force [6]. Several studies [7-9] have revealed that insertion of mini-screws at angles lesser or greater than 90° to the alveolar bone might decrease the anchorage stability of the mini-screw, as an oblique angle leads to a longer cantilever arm, causing mini-screw failure [8]. On the contrary, a few studies [10-12] have implied that the placement of mini-screws obliquely at an angle of 45° to 70° increased the primary stability owing to the greater area of the implant-cortical bone interface. Hence, there still exists an ambiguity in determining the ideal angle of insertion, balancing the cortical bone thickness, insertion torque of the implant, cortical bone stress, and primary stability of mini-screws while bringing about the necessary tooth movement without undue anatomical hindrance.

However, it is not feasible to assess the stress patterns generated on the viable tissues during the intrusion of mandibular incisors using mini-implants under clinical conditions. The simulation of biological tissues through finite element analysis works by reproducing a 3D virtual panorama to assess the biomechanical stress patterns and displacements in both static and dynamic conditions. Hence, this finite element study (FES) aims to evaluate and compare the efficiency of true intrusion and the level of stress produced in the PDL during the intrusion of four- and six- lower anterior teeth using bilateral mini-screws placed at three different angulations of 90° and 60°and 30° in relation to the bone surface directed apically.

## Materials and methods

Construction of finite element models

**Figure 1:**
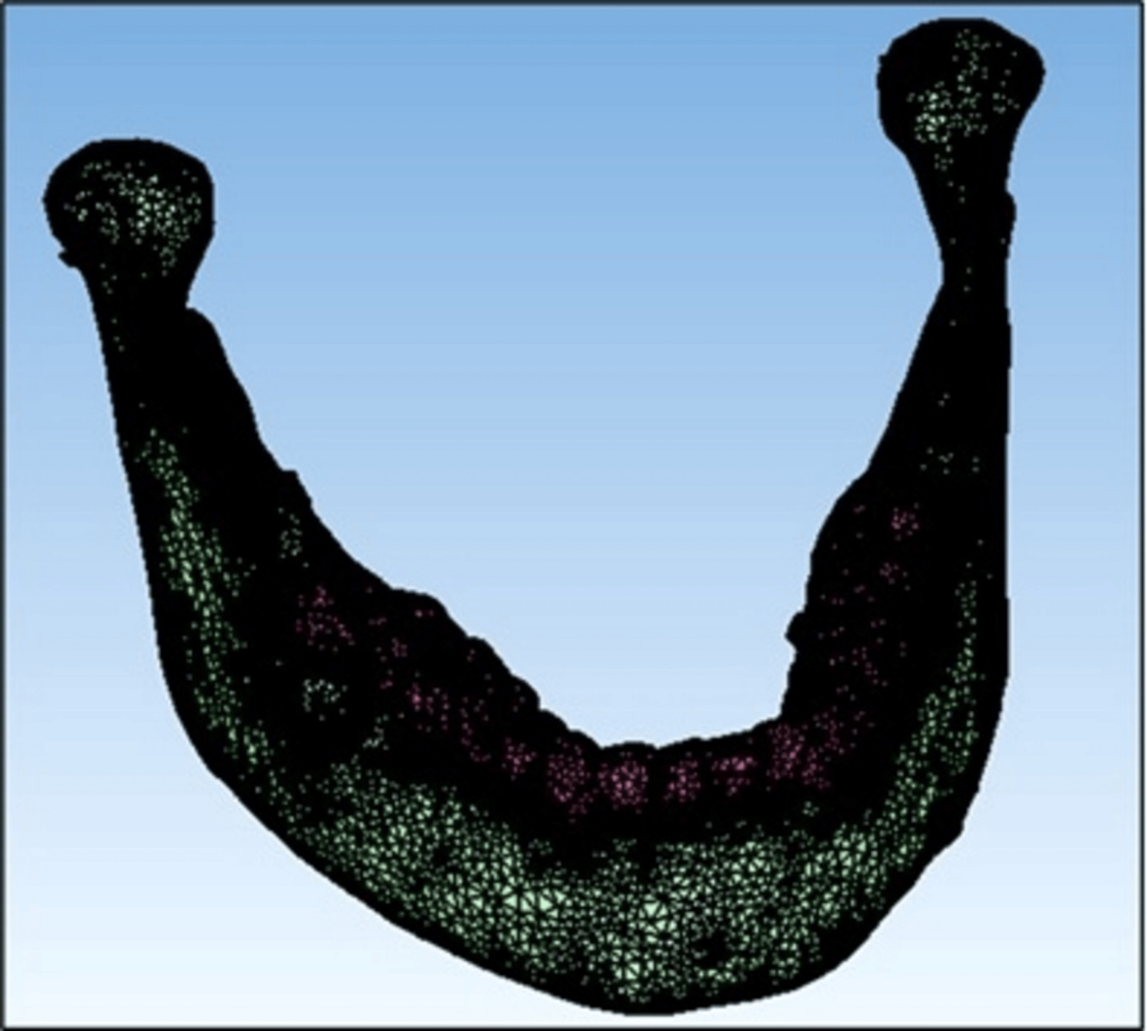
Finite element mesh simulation of the mandible consisting the teeth, periodontal ligament, and alveolar bone using Hypermesh v11 software.

**Figure 2:**
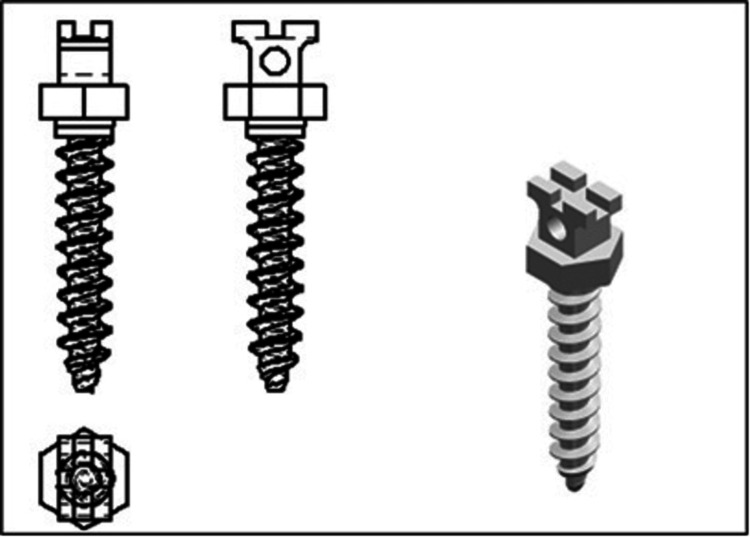
A conical, self-drilling miniscrew with a length of 6 mm and a diameter of 1.6 mm.

A cone-beam CT section of a well-aligned mandible with supra-erupted lower anterior teeth was imported to create a 3D finite element model using Standard Tessellation Language (STL) software [13]. The 3D data were updated with Hypermesh v11 software to create a tetrahedral finite element mesh, and a cloud of points was generated. Teeth, alveolar bone, and the PDL were all thought to be homogeneous and isotropic (Figure 1). A shell of 0.25-mm thickness surrounding every root of all six teeth was designed to act as PDL. The manual design of the mini-screw was simulated based on a conical, self-drilling device with a length of 6 mm and a diameter of 1.6 mm (Figure 2). MBT prescribed 0.022 x 0.028-in slot metallic brackets with 0.021 x 0.025-in stainless steel segmental lower arch wire that were attached to the teeth so that the midpoint of the brackets overlapped the facial axis point on the surface of the crowns. Boolean subtraction operations were performed to simulate accurate drilling holes necessary for mini-screw insertion. All materials in this study were assumed to be linearly elastic, homogeneous, and isotropic.

Mechanical properties

The mechanical properties of tissues and orthodontic appliances, including elastic modulus and Poisson’s ratio, were incorporated based on published data (Table 1) [14].

**Table 1:** Young’s modulus and Poisson’s ratio of various materials.

Structure	Young’s modulus (GPa)	Poisson’s ratio
Trabecular bone	1.5	0.30
Cortical bone	14.7	0.30
Tooth	20.7	0.30
Miniscrew (Ti6Al4V)	114	0.34
Periodontal ligament (linear model)	6.89 × 10^-5^	0.45
Stainless steel	193	0.29

Model categorization

**Figure 3:**
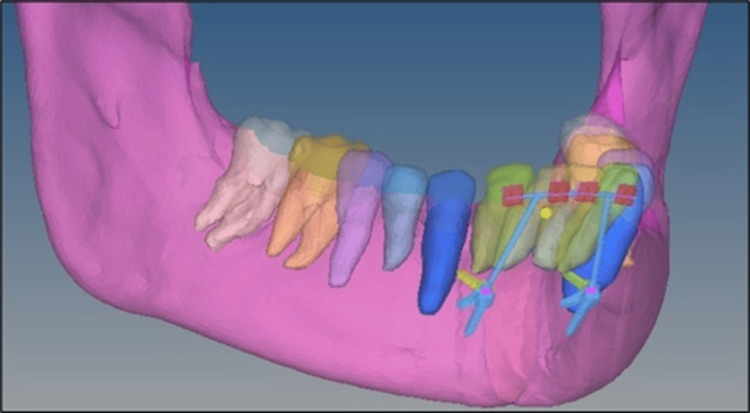
Intrusion of four anterior teeth with miniscrews placed distal to the lateral incisors at three different angulations 10 mm below the cementoenamel junction with the site of force application between the central and lateral incisor.

**Figure 4:**
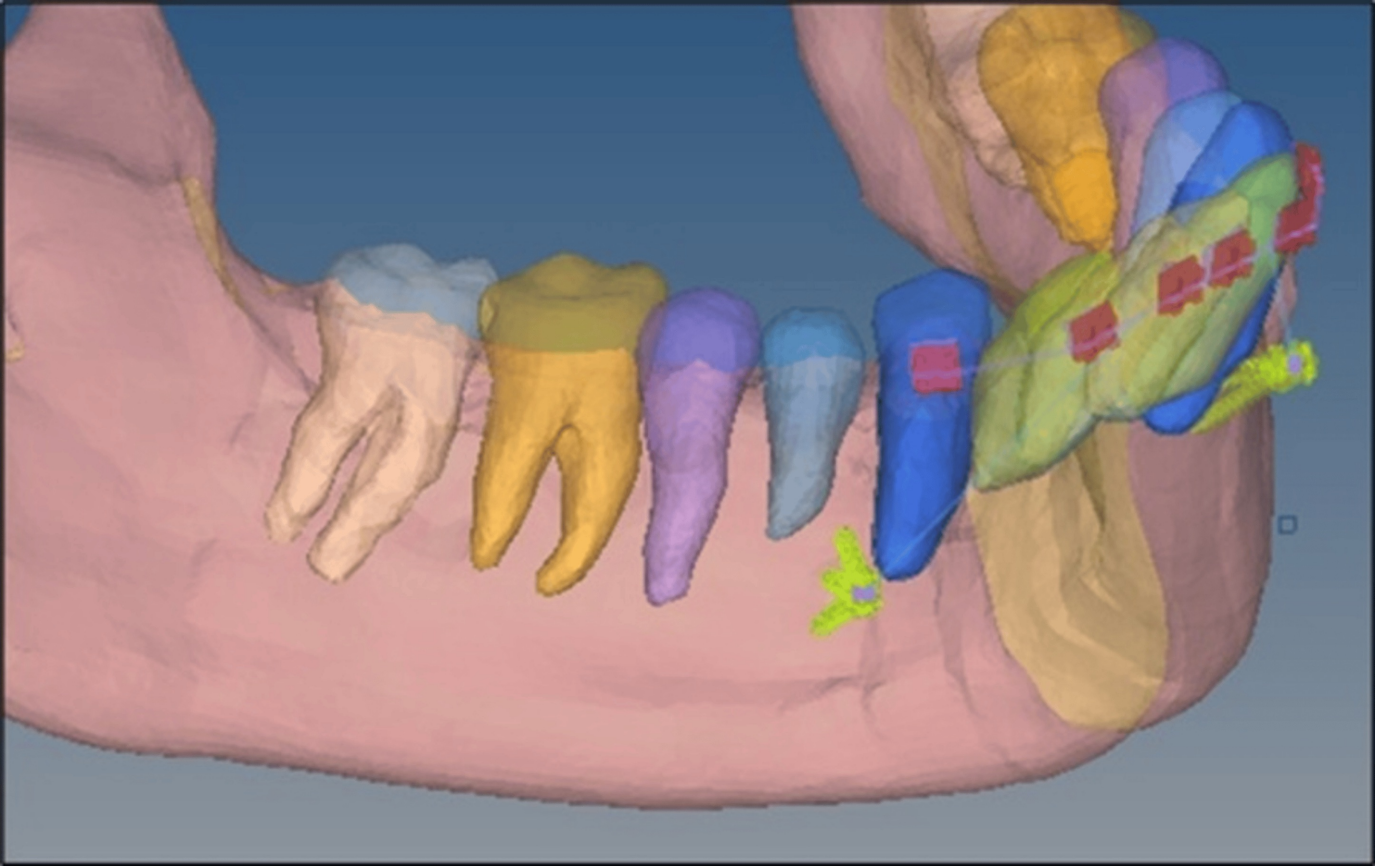
Intrusion of six anterior teeth with miniscrews placed distal to the canines at three different angulations 10 mm below the cementoenamel junction with the site of force application between the central and lateral incisor.

Model 1: Two mini-implants were placed between the lateral incisor and canine at 10 mm from the cementoenamel junction at an angle of 90° for the intrusion of four lower anterior teeth (Figure 3). Model 2: Two mini-implants were placed between the lateral incisor and canine at 10 mm from the cementoenamel junction at an angle of 60° for the intrusion of four lower anterior teeth (Figure 3). Model 3: Two mini-implants were placed between the lateral incisor and canine at 10 mm from the cementoenamel junction at an angle of 30° for the intrusion of four lower anterior teeth (Figure 3). Model 4: Two mini-implants were placed distal to the canine at 10 mm from the cementoenamel junction at an angle of 90° for the intrusion of six lower anterior teeth (Figure 4). Model 5: Two mini-implants were placed distal to the canine at 10 mm from the cementoenamel junction at an angle of 60° for the intrusion of six lower anterior teeth (Figure 4). Model 6: Two mini-implants were placed distal to the canine at 10 mm from the cementoenamel junction at an angle of 30° for the intrusion of six lower anterior teeth (Figure 4).

Quantitative model details

A mesh sensitivity analysis was performed to ensure solution accuracy and mesh independence. Progressive mesh refinement was performed while keeping all material properties, boundary conditions, and loading parameters constant. The stabilization of von Mises stress and displacement values with less than 5% deviation between successive refinements confirmed mesh convergence, and the optimized mesh was used for subsequent analyses. Table 2 displays the quantity of nodes and elements within each group.

**Table 2:** Quantity of nodes and elements within each group.

Scenario		Number of elements	Number of nodes
Mandibular four teeth	90°	328,273	476,234
	60°	329,981	477,568
	30°	331,016	479,273
Mandibular six teeth	90°	332,652	478,299
	60°	333,845	479,659
	30°	335,702	480,164

Simulation of force vector

Force vectors were attached to the region between the central and lateral incisors bilaterally for all six scenarios. Intrusion forces of 50 g (for four teeth) and 100 g (for six teeth) were applied on the segmental archwires, according to classical guidelines by Burstone [2].

Boundary conditions and the 3D coordinate system

The 3D coordinates were based on the occlusal plane. X-axis: Buccal (+) to palatal (−) direction; Y-axis: Anterior (+) to posterior (−) direction; Z-axis: Extrusion (+) to intrusion (−) direction. Non-linear static analysis was performed using ANSYS version 18.1 software.

Statistical analysis

In FES, validation of the analysis results using finite element simulations alone, rather than experimental data, is considered sufficient. Therefore, statistical analysis was deemed unnecessary.

## Results

The von Mises stress level in the periodontal ligament

**Figure 5:**
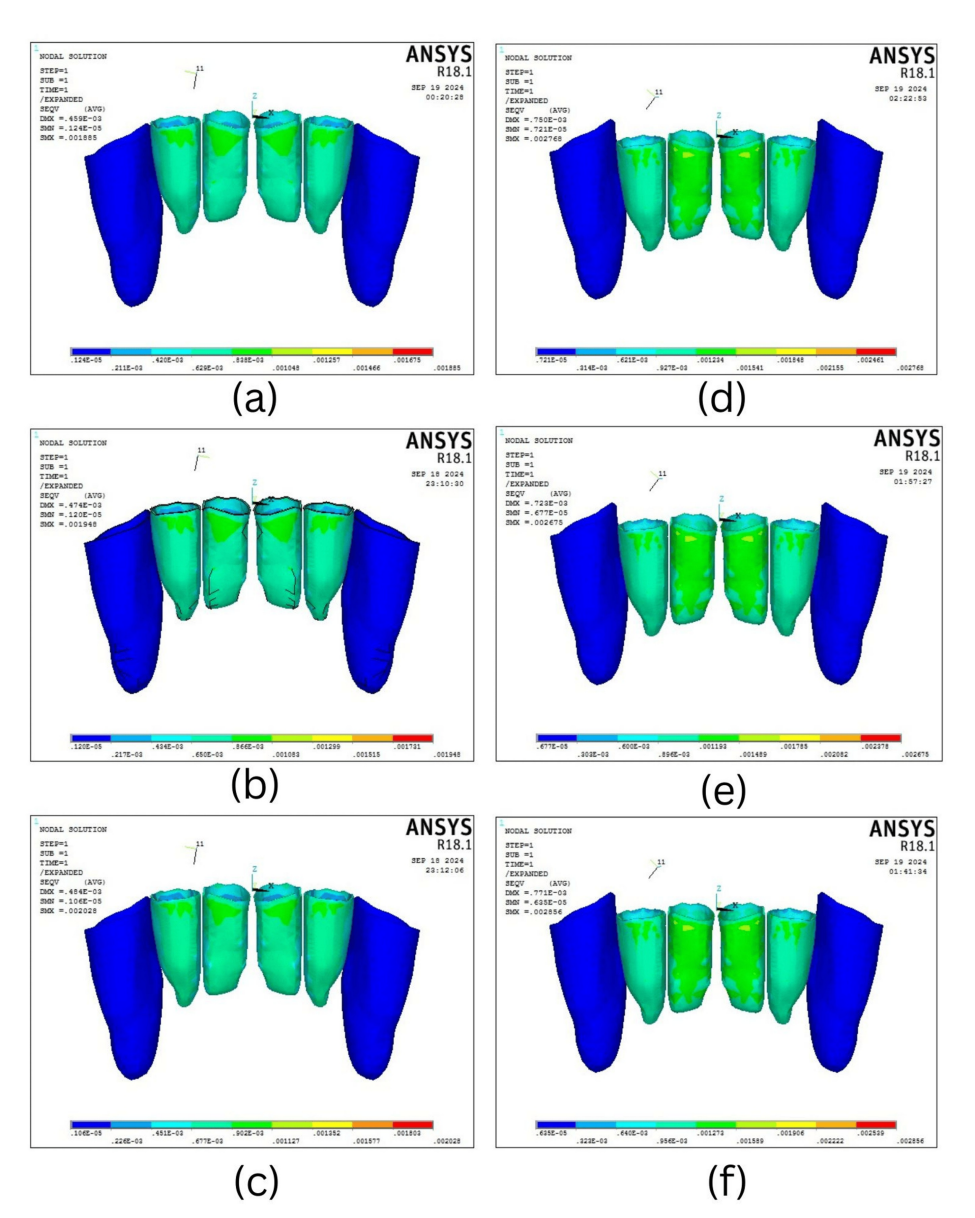
von Mises stress distribution in periodontal ligament (PDL) in four-teeth scenario when the mini-screw is placed at an angle of (a) 30°, (b) 60°, and (c) 90° and in six-teeth scenario when the mini-screw is placed at an angle of (d) 30°, (e) 60°, and (f) 90°.

The von Mises stress in the PDL during intrusion increased as mini-screw angulation decreased. In the four-teeth scenario, the highest stress was at 30° (0.002028 MPa), followed by 60° (0.001948 MPa), and the lowest at 90° (0.001885 MPa). Similarly, six-teeth intrusion showed maximum stress at 30° (0.002856 MPa) and minimum at 90° (0.002675 MPa) (Table 3, Figure 5). In the four-teeth scenarios, the maximum stress was concentrated at the cervical third of the central incisors, followed by the lateral incisors. Whereas, in the six-teeth scenario, the stress concentration was uniformly distributed and higher at the central incisor region, followed by the lateral incisor, with the least stress concentration at the canine region.

**Table 3:** Maximum von Mises stresses observed in the periodontal ligament (PDL).

Scenario	Insertion angle (°)	Maximum von Mises stress values in PDL (MPa)
Four-teeth scenario	90	0.001885
	60	0.001948
	30	0.002028
Six-teeth scenario	90	0.002675
	60	0.002768
	30	0.002856

Labio-lingual displacement along the y-axis

**Figure 6:**
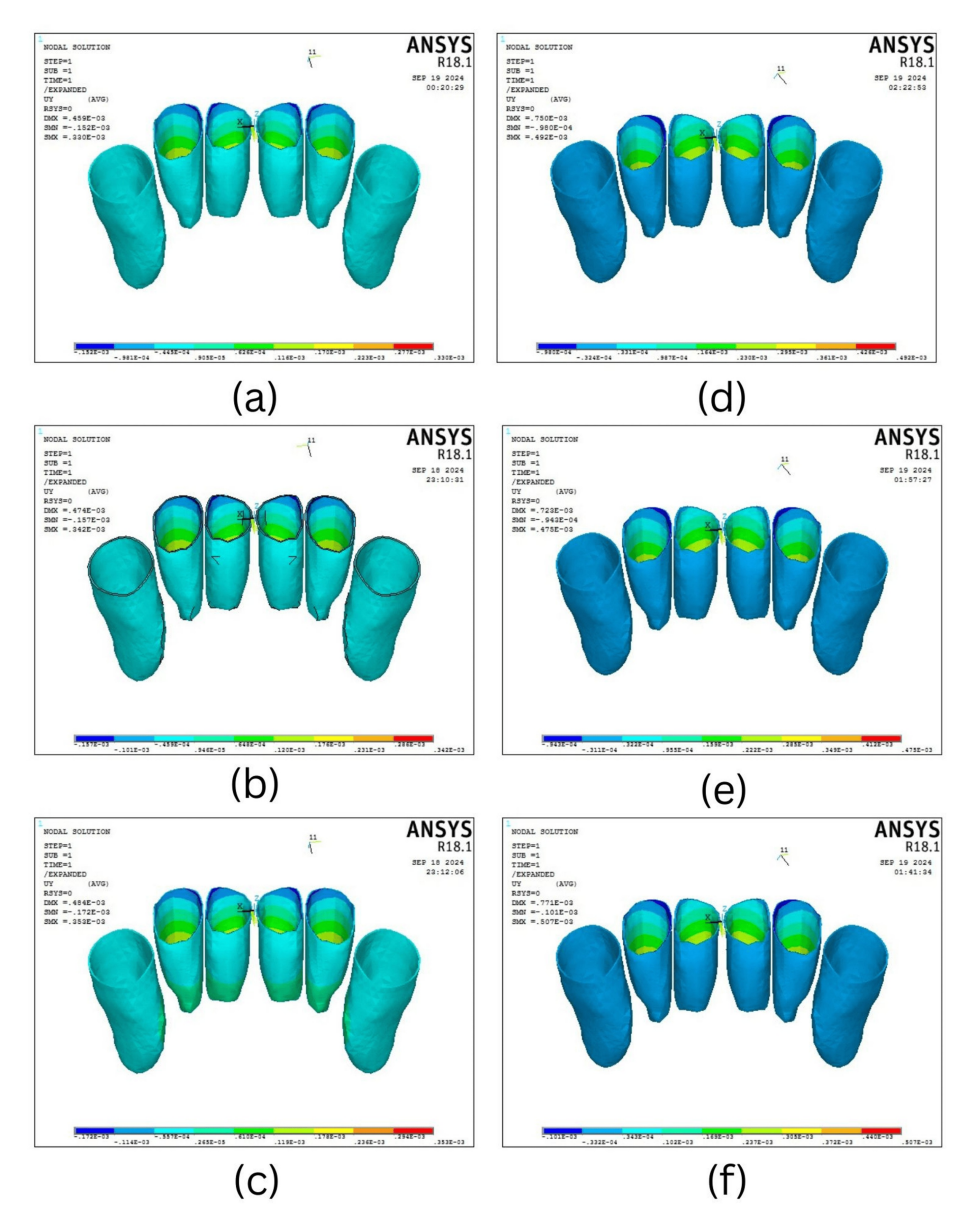
Buccolingual displacement in the four-teeth scenario when the mini-screw is placed at an angle of (a) 30°, (b) 60°, and (c) 90° and in the six-teeth scenario when the mini-screw is placed at an angle of (d) 30°, (e) 60°, and (f) 90°.

The labio-lingual displacement for the four-teeth scenario at the y-axis showed labial tipping of 0.000353 mm at 90°, 0.000342 mm at 60°, and 0.000330 mm at 30°, and the six-teeth scenario revealed labial tipping of 0.000507 mm at 90°, followed by 0.000492 mm at 60° and 0.000475 mm at 30° (Table 4, Figure 6).

**Table 4:** Labio-lingual displacement of teeth along the y-axis.

Scenario	Insertion angle (°)	Labial displacement (mm)	Lingual displacement (mm)
Four-teeth scenario	90	0.000353	-0.000172
	60	0.000342	-0.000157
	30	0.000330	-0.000152
Six-teeth scenario	90	0.000507	-0.000101
	60	0.000492	-0.000098
	30	0.000475	-0.0000943

Intrusive displacement along the z-axis

**Figure 7:**
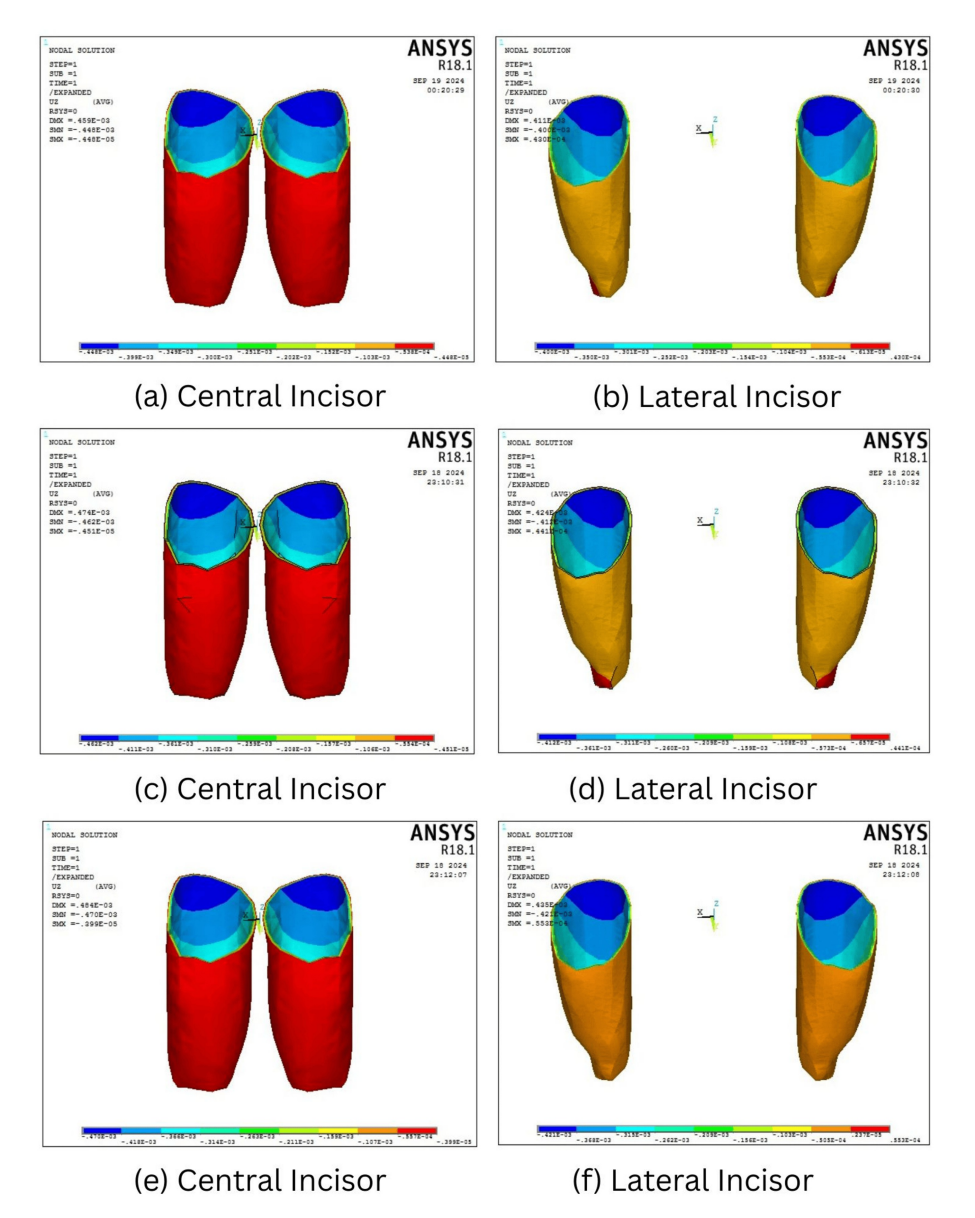
Amount of Intrusion in the four teeth scenario when the mini-screw is placed at 30° (a, b), 60° (c, d), and 90° (e, f).

**Figure 8:**
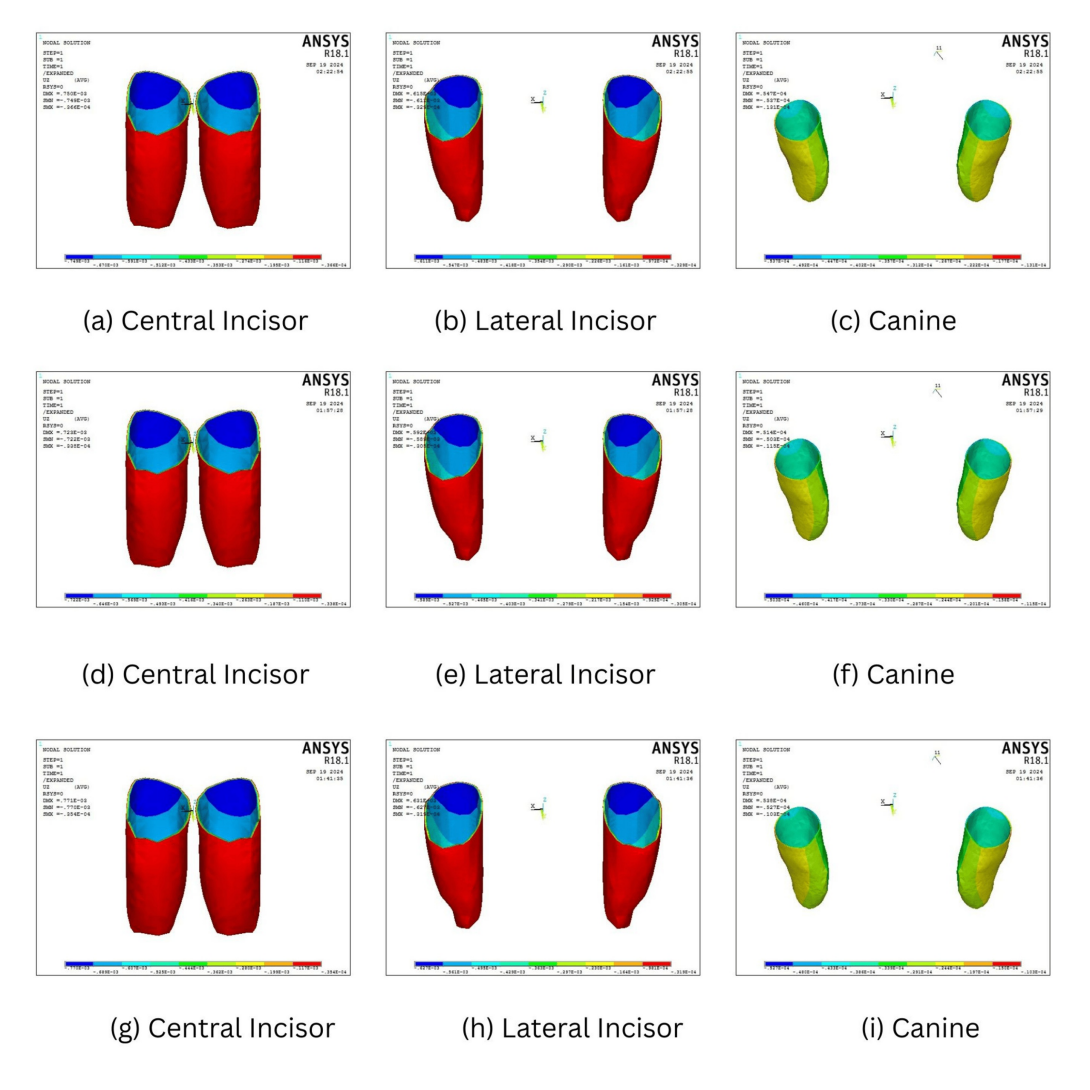
Amount of Intrusion in the six-teeth scenario when the mini-screw is placed at 30° (a-c), 60° (d-f), and 90° (g-i).

The intrusive displacement along the z-axis elicited maximum intrusion of teeth when the mini-screw was inserted at 90° and decreased progressively at 60° and 30° in both four- and six-teeth scenarios. The maximum intrusion was elicited in central incisors, followed by lateral incisors when mini-screws are placed at an angle of 90° in both four- (0.000470 mm and 0.000421 mm) and six-teeth (0.00077 mm and 0.000627 mm) scenarios. The canines experienced maximum intrusion of 0.0000537 mm when the mini-screws were placed at 60°, when compared to 90° (0.0000527 mm) and 30° (0.0000503 mm). Overall, 30° placement resulted in the least intrusive displacement across all teeth (Table 5, Figures 7, 8).

**Table 5:** Inciso-apical displacement of individual tooth along the z-axis.

Scenario	Insertion angle (°)	Central incisor (mm)	Lateral incisor (mm)	Canine (mm)
Four-teeth scenario	90	0.000470	0.000421	0
	60	0.000462	0.000412	0
	30	0.000448	0.000400	0
Six-teeth scenario	90	0.000770	0.000627	0.0000527
	60	0.000749	0.000611	0.0000537
	30	0.000722	0.000589	0.0000503

## Discussion

Deep bite correction using mandibular intrusion is the most commonly used technique to correct deep bites associated with exaggerated curve of spee, especially in Class II cases. Conventional intrusion-arch mechanics frequently cause labial tipping of the incisors or extrusion of molars, which does not always lead to favorable treatment outcomes. Hence, the use of mini-screws was popularized due to their effectiveness in achieving better bodily incisor intrusion, as the point of force application is closer to the center of resistance.

This study simulated 1.6 × 6 mm mini-screws, bilaterally placed at 10 mm from the cementoenamel junction with disto-intrusive force vectors to achieve a bodily intrusion with the placement of mini-screws as close to the center of resistance of four and six anterior mandibular teeth. This was in accordance with the results of McGrath et al., who showed true intrusion of six mandibular anterior teeth using bilateral mini-screws placed distal to canines at 90°, and for four incisors with implants distal to lateral incisors [14]. Moreover, Shalchi et al. recommended placement between the lateral incisor and canine, 8-11 mm from the cementoenamel junction, as a region with ideal bone thickness as well as in avoiding anatomical hindrance [15].

The evaluation of von Mises stress generated at the PDL revealed an increasing level of stress as the angle decreased from 90° to 30° in both four- and six-teeth scenarios, which aligned with the results of several studies [7,16,17]. According to Sharma et al., this may pertain to the increased area of cortical bone interface on oblique insertions, whereas, at 90°, there is a greater penetration of the mini-screw into the cancellous bone, which has the ability to absorb and dissipate the residual stresses generated during insertion of mini screw [16]. Raji et al. attributed this to the lesser insertion torque at 90° as the force is aligned more optimally with the natural orientation of trabeculae of the bone at perpendicular insertion, resulting in more efficient load transfer and lower stress concentrations [17].

Stress generated at insertion is an important factor in mini-implant stability, as increased stress might draw more cytokines, macrophages, and inflammatory mediators to the implant site, possibly resulting in a higher risk of mini-implant failure through loss of primary stability [18]. Nevertheless, the primary stability is not only dictated by the stress concentration alone but also by the surface area of interlocking between the mini-implant and the cortical bone [9]. Likewise, the inter-radicular approximation can be avoided with minimal oblique insertion. The stress levels measured at 60° insertion revealed slightly higher values than 90°, although not to a considerable extent. This was consistent with the results of a study by Zhao et al., which concluded that the best stability of mini-screws was achieved at a placement angle of 50° to 70° [19]. Both very oblique and vertical placement angles resulted in reduced stability of loaded mini-screws. In addition, a study by Woodall et al. revealed no significant differences in the anchorage resistance between 90° and 60° [8].

The labial tipping observed in all six scenarios revealed increasing tipping as the angle increased from 30° to 90°. This can be biomechanically attributed to the greater approximation of the head of the mini-screw toward the long axis of the teeth in oblique insertion, thereby directing a bodily force vector [8]. Hence, a greater labial tip was generated at 90° when compared to oblique mini-screw insertion. This provides less stable post-treatment retention owing to the untenable inclination of the lower incisors with the basal bone, with a high risk of relapse [20].

The evaluated intrusive displacement showed a consistent reduction as the mini-screw angulation decreased from 90° to 30°, which is attributed to the increasing proximity of the mini-screw head to the center of resistance. This parallels the above findings that an increased sagittal distance of the mini-screw from the center of resistance enhances both intrusive and labial moments. Nevertheless, canines showed maximum intrusion at 60°, and the incisors elicited a good amount of overall intrusion at 60° when compared with 90°. Despite angulation changes, all six scenarios showed maximum intrusion on central incisors, followed by the lateral incisor and canine, aligning with the study by Agarwal et al., likely due to their greater horizontal distance from the implant, enhancing intrusive moment on the farthest teeth [21].

This FES has limitations due to reliance on input accuracy, overlooking individual anatomical and biological variability, and inability to fully replicate dynamic clinical responses and tissue behavior in simulations. Nevertheless, the choice of insertion angle and the location of mini-implant placement based on cortical thickness should aim to achieve the required stability while minimizing stress on the surrounding bone. Selecting the appropriate insertion angle involves considering the anatomical constraints of the implantation site. In areas with limited space between adjacent roots, a more oblique insertion direction may be advantageous to minimize the risk of root contact.

## Conclusions

The choice of the insertion angle and the location of mini-implant placement based on cortical thickness should aim to achieve the required stability while minimizing stress on the surrounding bone. Considering the findings of the present study, the oblique placement of bilateral mini-screws is recommended with a disto-intrusive force vector to achieve a relatively true intrusion of four- and six- mandibular anterior teeth with minimal labial tip with lesser stress generated at the PDL on the insertion of mini-screws.
